# Quantitative Analysis of Boron Trichloride Using ^11^B Quantitative Nuclear Magnetic Resonance Spectroscopy (qNMR)

**DOI:** 10.1155/jamc/4351793

**Published:** 2026-10-25

**Authors:** Daniel Van Niekerk, Jaspaul Tatlay, Hilaire V. Kemami Wangun, Nicholas J. Morehouse

**Affiliations:** ^1^ Analytical Development Gilead Sciences 1021 Hayter Road Edmonton T6S 1A1 Alberta Canada gilead.com

## Abstract

Boron trichloride (BCl_3_) is highly reactive and degrades rapidly when exposed to moisture, complicating analyses, and posing safety risks under standard laboratory conditions. To address these challenges, quantitative ^11^B nuclear magnetic resonance (qNMR) spectroscopy has been introduced as an analytical method to accurately quantitate boron trichloride in solution. This technique involves the reaction of boron trichloride with methanol to produce trimethyl borate, which is then quantified using an external standard. The preparation process allows for the reaction and sample preparation to occur in a single step within the same vial, ensuring a safe, fast, and reliable analysis. The ^11^B qNMR method was validated, demonstrating linearity (0.02 M to 0.50 M with *r* = 0.9999), accuracy (recovery of 100.05%), precision (inter‐ and intraday), stability, and robustness. This paper highlights the effectiveness of the ^11^B qNMR method for the accurate, reliable, fast, and safe quantification of boron trichloride in organic solvents such as dichloromethane.

## 1. Introduction

Boron trichloride (BCl_3_) is a boron halide and a strong Lewis acid that is widely used in organic synthesis [1]. Its versatility spans several key transformations, including ether cleavage, hydroboration of alkenes, arene borylation, methyl ester formation from fatty acids, and transesterification of triglycerides [1–7]. Commercially, boron trichloride is available as a colorless liquid or gas under pressure, often in solution with an organic solvent (e.g., dichloromethane, heptane, toluene, etc.) to facilitate handling. Despite its utility, boron trichloride poses significant challenges in analytical settings due to its high reactivity. Upon exposure to moisture or protic solvents, it rapidly decomposes with the release of hydrogen chloride making it hazardous and difficult to manage in standard laboratory environments. Therefore, a safe and reliable analytical method is required to accurately quantify boron trichloride for organic synthetic applications where accurate stoichiometric additions are required.

Quantitative determination of boron trihalides is commonly achieved through titration, either directly or following hydrolysis to yield hydrogen halides and boric acid [8–11]. Colorimetric titration employing *N*, *N*‐dimethylaniline has been proposed as a benchtop approach for boron trihalide analysis [9, 10] and has been specifically investigated for boron trichloride [10]. However, titration‐based quantification of boron trichloride remains challenging due to its high reactivity and associated handling hazards, including its volatility, vigorous and exothermic hydrolysis, and the need for specialized apparatus and controlled environments. Analytical procedures must therefore be conducted under strictly anhydrous and inert conditions to prevent hydrolysis. Despite its industrial importance, comprehensive and validated quantitative methodologies for boron trichloride remain limited in the literature.

Boron nuclear magnetic resonance spectroscopy (NMR) was postulated as a potential alternative analytical method for the quantitation of boron trichloride. The application of boron NMR for the determination of structures for compounds with boron skeletons is well established in the literature [12]. NMR spectroscopy is an attractive method for quantification because the integral of each peak in the acquired spectrum is directly proportional to the number of nuclei of the compound present in solution [13]. Boron has two stable spinning isotopes, ^10^B and ^11^B, that could be used in NMR [12]. For sensitivity, ^11^B is the nucleus of choice due to its higher natural abundance (80.1%), higher gyromagnetic constant, and lower quadrupolar moment [12, 13]. A major advantage with ^11^B NMR is that no nuclei other than boron are detected in this technique, resulting in very little interference [13].

Quantitative NMR (qNMR) is a well‐established technique in analytical chemistry, routinely employed for the accurate quantification of active pharmaceutical ingredients, impurities, and organic components [14–18]. Numerous studies have demonstrated that the accuracy and precision of qNMR are comparable to those of analytical liquid chromatographic methods [18–21]. In addition to being inherently quantitative, qNMR is nondestructive, enables short analysis times, and allows the quantification of analytes in multicomponent mixtures within a single experiment.

Typically, a structurally distinct internal standard is used for qNMR within the same solution as the analyte. Selecting a suitable internal standard for qNMR is not always trivial as there are various factors that need to be considered. These factors include solubility of the standard in the NMR solvent, compatibility with the analyte of interest, and distinct chemical shift differences of the signal in the spectra (i.e., no overlapping of the chemical shifts for the internal standard and the analyte) [18, 19]. Due to the reactivity of boron trichloride, the selection of an appropriate internal standard for a ^11^B qNMR method would be a major challenge. Therefore, a qNMR method that uses an external standard for the quantitation of boron trichloride could be a more appropriate alternative [18–20]. An external standard qNMR method involves using the same volume of analyte and external standard solutions placed in separate NMR tubes and analyzed under the exact same instrument conditions [22–24]. Comparable precision and accuracy between an internal standard and an external standard qNMR method have been reported in the literature [22, 25]. A quantitative ^11^B qNMR method using an external standard has been developed and validated in the literature for the determination of boric acid in commercial biocides [13].

This study presents the development and validation of a safe, accurate, and robust ^11^B qNMR method for quantifying boron trichloride in dichloromethane. The approach involves converting boron trichloride into trimethyl borate (stable and less hazardous derivative) prior to analysis, thereby mitigating handling risks. Comprehensive validation was performed to confirm the reliability of both the sample preparation and the qNMR quantification methodology.

## 2. Materials and Methods

### 2.1. Chemicals and Materials

1.0 M boron trichloride in dichloromethane was obtained from an undisclosed commercial site and the certificate of analysis was provided as part of the purchase. Trimethyl borate was purchased from Sigma‐Aldrich (purity ≥ 99.5%), deuterated methanol (MeOH‐*d*_4_) was purchased from Cambridge Isotope Laboratories (*D* ≥ 99.8%) and thin‐wall natural quartz NMR sample tubes (< 0.1 ppm boron) were purchased from Norell. Standard borosilicate NMR tubes are known to interfere with ^11^B NMR spectra; this effect can be minimized by using natural quartz tubes, which contain negligible boron (< 0.1 ppm) and are therefore preferred for quantitative analyses [13]. Due to their higher cost compared to borosilicate tubes, a washing protocol was developed to enable reuse. Natural quartz tubes are more fragile than borosilicate tubes, requiring careful handling during cleaning. The procedure involved decanting the contents, rinsing three times with methanol, followed by three rinses with acetone, and drying at 60°C. The effectiveness of this cleaning process was evaluated by ^11^B NMR analysis of washed tubes filled with MeOH‐*d*_4_ to assess carryover or interference. This protocol may be more rigorous than necessary and could be further optimized. It is highly recommended to evaluate tube quality prior to conducting external standard qNMR quantitation due to potential tube‐to‐tube volume variability [17, 26]. Evaluation of the natural quartz NMR sample tubes was performed as per Ochoa et al. and was deemed suitable for the intended application [26].

### 2.2. Standard and Sample Preparations

The concentrations of the external standard and boron trichloride sample were optimized to enable accurate qNMR analysis at a target molarity of approximately 1.0 M. The external standard qNMR method requires equal volumes of analyte and standard solutions, placed in separate NMR tubes and analyzed under identical conditions. A calibrated positive displacement pipette was used to dispense 700 μL of each solution volumetrically into quartz NMR tubes, ensuring consistent sample volume, a critical parameter for quantitative accuracy [13].

The external standard was prepared by accurately preparing trimethyl borate in MeOH‐*d*_4_ solution with a final molarity of 0.10 M. Trimethyl borate external standard was prepared by accurately pipetting 5.0 mL of deuterated methanol (MeOH‐*d*_4_) into a 20‐mL scintillation vial, followed by the addition of 55.5 μL of trimethyl borate. The resulting solution was thoroughly mixed to ensure homogeneity. The masses of trimethyl borate and deuterated methanol were calculated by correcting for the respective densities (0.932 g/mL for trimethyl borate and 0.888 g/mL for MeOH‐*d*_4_). This methodology was chosen for its ease of preparation and demonstrated good repeatability and reproducibility. Alternatively, the deuterated methanol and trimethyl borate can be accurately weighed using these volumes. Two NMR tubes were prepared by accurately transferring 700 μL aliquots of the standard solution into quartz NMR tubes, which were then capped.

Optimal sample preparation was found with a 1:9 dilution resulting in a final sample concentration of approximately 0.10 M for boron trichloride converted to trimethyl borate. The sample was prepared by accurately pipetting 9.0 mL of MeOH‐*d*_4_ into an empty 20‐mL scintillation vial. The weight of the MeOH‐*d*_4_ and scintillation vial with cap was recorded. Approximately 1 mL of the boron trichloride sample (1.0 M BCl_3_ in dichloromethane) was carefully added to the scintillation vial containing the 9.0 mL of MeOH‐*d*_4_. Boron trichloride is highly reactive to moisture and should be handled in an inert atmosphere. The weight of the resulting solution in the scintillation vial with cap was recorded, and the weight of the added 1.0 M boron trichloride in dichloromethane was determined by difference. The resulting sample solution was thoroughly mixed to ensure reaction completion and solution homogeneity. The sealed sample solution was left to equilibrate to room temperature. A 700‐μL aliquot of the sample solution was accurately transferred into a quartz NMR tube and capped. Duplicate sample solution preparations were made for all quantitative analyses. To evaluate reproducibility, two independent sample preparations (*n* = 2) were performed. An acceptance criterion of an absolute difference of ≤ 3% between preparations is recommended to ensure consistency and reliability of the analytical results.

### 2.3. NMR Instrument Conditions and Processing

All ^11^B NMR experiments were acquired at 298 K using a Bruker Avance III 400 MHz spectrometer. The experiments were conducted using a predefined 90° pulse program (B11 ZG) without spinning. NMR spectra were collected using the following acquisition parameters (standard parameters of B11 ZG): number of FID data points (TD) = 64 K, acquisition time (AQ) = 1.2 s, and spectral width = 25,510 Hz. The relaxation delay (*D*1) was set to 1 s, and the number of scans (NS) was set to 128 scans.

All NMR data were processed using TOPSPIN software. Processing steps included manual phase correction, automatic baseline correction, and manual integration. The absolute integral value (Integral [abs]) was used to quantify the amount (molarity) of boron trichloride in the samples using equation (1). Equation (1) uses an absolute quantitation method where the main component can be calculated directly using an external standard of known content [17, 18]. Equation (1) requires the concentration (*C*_Std_) of the external standard in molarity, average integral value of the peaks of the two standard solutions (*I*_Std_), the integral value of the peak in the sample solution (*I*_Sample_), and the number of contributing nuclei of the signals for the sample (*n*_Sample_) and standard (*n*_Std_). This approach assumes precise and equal volumes of sample and standard (700 μL each) in quartz NMR tubes.

(1) Molarity of BCl3 in sample mol/L=CStd×ISampleIStd×nStdnSample×Dilution Factor.

Equation (1) is analogous to external standard methods described in the qNMR literature, such as PULCON [18, 23, 25, 27]. In this study, additional PULCON parameters (e.g., pulse length, temperature) were not applied because both standard and sample were analyzed in the same sequence under identical conditions, minimizing their impact. Acquisition time was approximately 5 min per analysis using current qNMR parameters.

The calculated amount of converted boron trichloride was corrected for the dilution factor (∼10) to yield the actual sample concentration (∼1.0 M) in equation (1). If precise volumetric addition of the boron trichloride solution is not feasible, the dilution factor can be calculated from the recorded mass and the known density of the 1.0 M boron trichloride solution in dichloromethane. The density of 1.0 M boron trichloride in dichloromethane (1.326 g/mL) is widely available in product specifications from chemical suppliers such as Sigma‐Aldrich, Thomas Scientific, and Fisher Scientific.

Finally, the sample spectrum should be examined to confirm complete conversion of boron trichloride to trimethyl borate, indicated by the presence of only the trimethyl borate signal (18.5 ppm).

## 3. Results and Discussion

### 3.1. Chemistry and NMR Development

Boron trichloride in dichloromethane is highly reactive, volatile, and difficult to handle under the inert conditions required for safe manipulation, making it cumbersome for standard analytical laboratories. Direct NMR analysis of boron trichloride poses significant safety risks, including tube breakage, hydrochloric acid off‐gassing, and disposal challenges. To obtain a more stable analyte that is less reactive and volatile, boron trichloride was reacted with deuterated methanol (MeOH‐*d*_4_) to form trimethyl borate. Methanol‐boron trichloride solvent reactions are well documented in the literature [1, 28]. The proposed reaction between boron trichloride and methanol is shown:

(2) BCl3+CH3OH⟶CH3O3B+3HCl.

Full conversion from boron trichloride to trimethyl borate was achieved stoichiometrically by using excess MeOH‐*d*_4_. This approach integrates reaction and sample preparation in a single step within the same vial. Methanol is fully miscible with dichloromethane with no phase separations, making it an ideal solvent for this method.

The resulting trimethyl borate was quantified against an external trimethyl borate standard of known concentration. Trimethyl borate is commercially available at high purity (> 99%) and serves as both reference and quantitation standard. The advantage of ^11^B NMR is that only boron‐containing analytes will be observed without any interferences from dichloromethane or other nonboron compounds [13, 18].

^11^B NMR spectra of the converted sample (trimethyl borate‐*d*_9_) and the external trimethyl borate standard were identical, each showing a single peak at 18.5 ppm (Figure 1). No additional boron signals were observed, confirming complete conversion of boron trichloride to trimethyl borate in the sample solution. The trimethyl borate ^11^B NMR spectrum corresponds to that recorded in the literature ([26] supporting information) [29]. This chemical shift agrees with literature values for trimethyl borate [29] and is distinct from boron trichloride (∼46.8 ppm) [3, 12], demonstrating method specificity. This study confirmed that the trimethyl borate chemical shift is independent of concentration, consistent with previous observations for ^11^B NMR of boric acid [13, 30, 31]. Therefore, conversion of boron trichloride to trimethyl borate in deuterated methanol proved to be an effective preparation strategy, yielding a stable sample suitable for accurate ^11^B NMR analysis.

**FIGURE 1:**
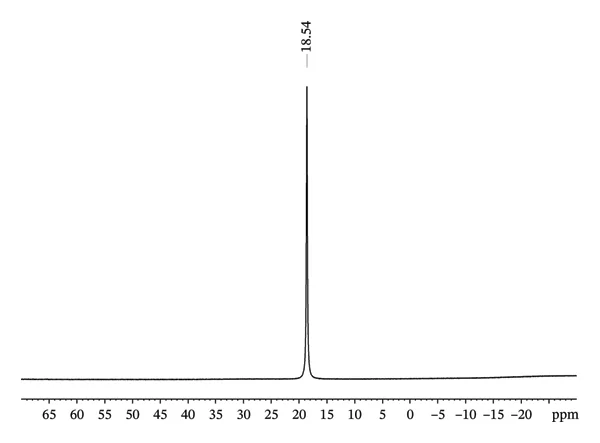
^11^B NMR spectrum of trimethyl borate (18.5 ppm).

As described in the experimental section, the external standard qNMR method requires equal volumes of analyte and external standard solutions, each placed in separate NMR tubes and analyzed under identical instrument conditions. During method development, the reproducibility and repeatability of volumetric dispensing, specifically 700 μL, were evaluated for both sample and standard preparations. In accordance with analytical method validation guidelines [17], a relative standard deviation (RSD) of ≤ 2% was achieved, demonstrating the precision and reliability of the volumetric transfer process. Further validation of the method confirmed its suitability for quantitative analysis, with consistent performance across independently prepared replicates.

The developed qNMR approach was confirmed to be fit‐for‐purpose for quantitation of boron trichloride using the described sample preparation and analytical parameters. The quantitation strategy, outlined in equation (1), is based on an absolute method of quantification. In cases where instrument‐related variability, such as fluctuations in temperature, pulse length, or receiver gain, is significant, these parameters may be incorporated into equation (1) following the PULCON methodology [18, 23, 27]. For analyses requiring extended linearity or broad concentration ranges, explicit correction for receiver gain in both standard and sample acquisitions is recommended. Under the current experimental conditions, however, the method demonstrated acceptable precision and reliability without additional correction factors. In situations where variability in analyte quantity is anticipated, a calibration curve can be employed for quantitative analysis [13, 16, 32].

### 3.2. NMR Method Optimization

Two critical parameters for optimizing qNMR acquisition are NS and relaxation delay (D1) [13]. The objective was to refine these parameters to ensure reliable quantitation at the target sample concentrations (approximately 0.1 M diluted) while minimizing analysis time. NS values of 32, 128, and 256 were evaluated across three independent preparations of the trimethyl borate standard. Figure 2 illustrates the relationship between the NS and absolute integration (average of the three preparations).

**FIGURE 2:**
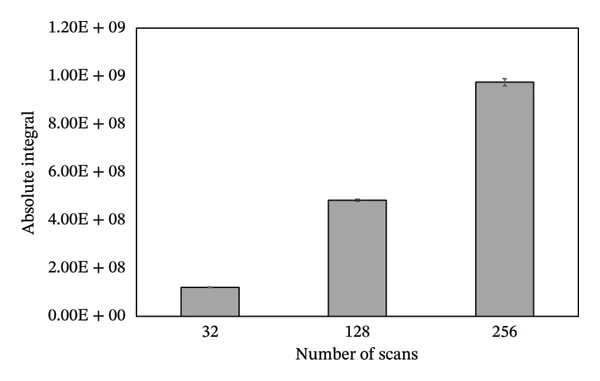
Effect of number of scans (NS) on absolute signal integration for ^11^B qNMR.

As expected, increasing the NS produced a linear increase in peak response (absolute integration), confirming proper performance of all NMR system components (RF amplifier, pulse generator, and radio frequency transmitter) and ensuring accurate, repeatable results [13]. The results indicate that the method can be further optimized for lower boron trichloride concentrations by increasing the NS. For the current concentration range, 128 scans provide sufficient signal intensity for accurate quantitation.

The boron nucleus is quadrupolar; consequently, longitudinal relaxation times (*T*_1_) for solution‐phase ^11^B NMR are typically very short and generally falling within the microsecond to millisecond range [13, 30]. To experimentally determine *T*_1_ under the conditions used in this study, inversion‐recovery experiments were performed at three concentrations (0.05, 0.10, and 0.20 M) using a 90° pulse angle. Across the concentrations evaluated, the measured *T*_1_ values were approximately 15 ms, indicating minimal concentration dependence.

For quantitative NMR measurements, a relaxation delay of approximately five times *T*_1_ is required to ensure complete longitudinal relaxation. Based on the experimentally determined *T*_1_, an initial relaxation delay (*D*1) of 1 s was selected. Previous literature reports indicate that a *D*1 as short as 0.1 s is sufficient for the quantitative determination of boric acid in deuterated water [13, 30]. To experimentally verify the suitability of the chosen relaxation delay, *D*1 values of 1, 5, and 30 s were evaluated using 128 scans across three independent preparations of the trimethyl borate standard. As shown in Figure 3, increasing the relaxation delay beyond 1 s resulted in no meaningful change in signal integration, confirming that a *D*1 of 1 s is sufficient for accurate quantitative analysis.

**FIGURE 3:**
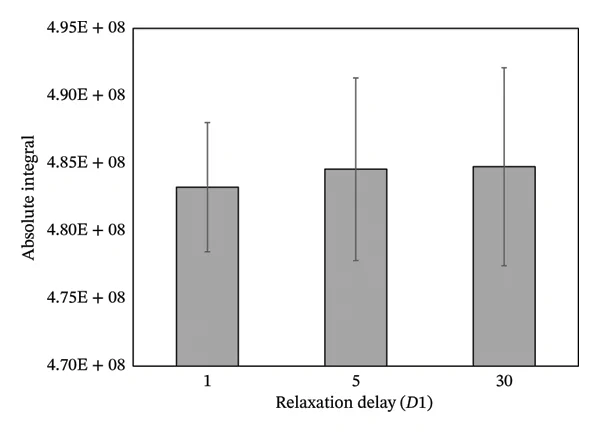
Effect of relaxation (D1) on absolute signal integration for ^11^B qNMR.

The selected parameters (NS = 128, *D*1 = 1 s) were appropriate for the intended concentration range, resulting in a method that is fast, accurate, and repeatable for routine boron trichloride analysis. These settings allowed an acquisition time of approximately 5 min, making this approach highly efficient for routine analysis. Further optimization may be required for lower concentrations, but the current settings provide robust performance for the proposed qNMR method.

### 3.3. Method Validation

#### 3.3.1. Selectivity, Linearity, and Range

The ^11^B qNMR method for the analysis of boron trichloride in dichloromethane was validated to ensure reliability for routine sample analysis. Recommended protocols for qNMR validation have been proposed in the literature [33, 34]. Validation parameters assessed in this study included selectivity, linearity, precision (intra‐ and interday), limit of detection (LOD), limit of quantitation (LOQ), and accuracy. The validation results are summarized in Table 1.

**TABLE 1:** Performance data of the ^11^B qNMR method.

Parameter	Result
Concentration range (M)	0.02–0.50
Correlation coefficient (r)	0.9999
LOD (M)	0.007
LOQ (M)	0.02

Selectivity was confirmed by analyzing a blank solution (MeOH‐*d*_4_), the trimethyl borate standard, and the boron trichloride samples. No signal was observed at the characteristic chemical shift of trimethyl borate (18.5 ppm) in the blank solution, confirming the absence of matrix interference. The boron trichloride sample prepared in MeOH‐*d*_4_ exhibited a signal at 18.5 ppm due to conversion to trimethyl borate, consistent with experimental and literature values [29]. No boron trichloride peak (∼46.8 ppm) was observed, as expected, due to rapid conversion to trimethyl borate in MeOH‐*d*_4_ [3, 12]. These results confirm selectivity for the intended analyte.

Linearity was evaluated over a concentration range of 0.02 to 0.50 M to confirm the linear relationship between ^11^B NMR response (absolute integration) and sample concentration (*M*). This method demonstrated excellent linearity with a correlation coefficient (*r*) of 0.9999, and a slope of 4.81 × 10^9^ (Figure 4). These results confirm a strong linear relationship between qNMR response and concentration.

**FIGURE 4:**
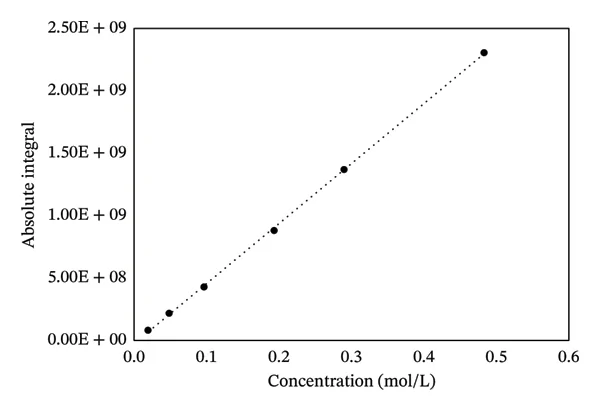
Linearity of trimethyl borate.

#### 3.3.2. Limits of Detection and Quantitation

LOD and LOQ were calculated according to ICH Q2 (R2), using the standard deviation of the regression *y*‐intercept (*S*_*a*_) and the slope (*b*) of the calibration curve, as per equations (3) and (4) [35].

(3) LOD=3.3×Sab,

(4) LOQ=10×Sab.

The resulting LOD and LOQ were 0.007 M and 0.02 M, respectively, demonstrating adequate sensitivity for the proposed qNMR method.

#### 3.3.3. Precision

Intraday and interday precisions of the proposed ^11^B qNMR method were evaluated by triplicate assays of trimethyl borate at three concentration levels (0.05, 0.1, and 0.2 M). Intraday precision was assessed at 0, 6, and 12 h, whereas interday precision was evaluated from day 0 to day 5. The low RSD values demonstrated good reproducibility of the method. The results are summarized in Table 2.

**TABLE 2:** Intraday and interday precision data for ^11^B qNMR method.

Parameter		Concentration (M)		
		0.05	0.1	0.2
Intraday	% Found	100.01	98.37	101.12
		98.67	98.37	100.91
		98.42	98.36	100.81
	Average	99.03	98.37	100.95
	± SD	0.8512	0.0079	0.1616
	% RSD	0.86	0.01	0.16
	Std. Error^a^	0.4914	0.0046	0.0933

Interday	% Found	100.01	98.37	101.12
		99.11	98.05	101.08
		98.15	98.62	101.07
		97.04	98.90	101.19
		96.97	98.40	101.14
		96.66	98.19	101.10
	Average	97.99	98.42	101.12
	± SD	1.3440	0.3044	0.0439
	% RSD	1.37	0.31	0.04
	Std. Error^a^	0.5487	0.1243	0.0179

^a^Standard error of the mean (SD/√*n*).

#### 3.3.4. Accuracy

Accuracy was assessed by calculating percentage recovery by comparing measured concentrations to nominal concentrations prepared by accurate weighing and dilution. Accuracy was evaluated by performing triplicate analyses of trimethyl borate at three concentration levels (0.05, 0.10, and 0.20 M). Percentage recoveries were calculated using equation (1). Accuracy data for the method are summarized in Table 3.

**TABLE 3:** Accuracy data for ^11^B qNMR method.

Amount prepared (*M*)	Amount found (*M*)	% Recovery^a^
0.05	0.05	99.75
0.1	0.10	100.46
0.2	0.20	99.93
Average		100.05
Standard deviation		0.369
%RSD		0.37

^a^Each result is the mean recovery of three separate determinations.

### 3.4. Quantitative Results

Quantitative ^11^B NMR analysis was performed to determine the molarity of boron trichloride following conversion to trimethyl borate, using the validated sample preparation procedure and an external standard approach. At controlled room temperature (∼25°C), both the standard and sample solutions remained stable over a 24 h period, with no observable change in molarity. The measured concentration of boron trichloride was consistent with the certificate of analysis provided by the commercial supplier, which reported a value of 1.02 M. The ^11^B qNMR results agreed with the vendor’s titration‐based analysis on the certificate of analysis (1.02 M), with recoveries of 95%–101% (mean 98%, *n* = 8). The method thus matches titrimetric accuracy while offering improved precision, a direct spectroscopic measurement, and safer handling.

A key objective of this method development was to enable long‐term stability assessment of commercial boron trichloride solutions in dichloromethane. To evaluate stability, a sample was monitored over 18 days under ambient conditions. Aliquots were collected on days 0, 5, 10, and 18 and analyzed using the validated ^11^B qNMR method. Molarity values across time points are summarized in Table 4. The results demonstrated adequate chemical stability, with an RSD of 1.8% across the study period. All measurements fell within the supplier’s specification range of 0.9500 to 1.100 M, confirming the reliability of the method for extended stability studies.

**TABLE 4:** Stability of commercial boron trichloride in DCM over 18 days.

Time point (days)	Molarity^∗^
0	1.00
5	1.01
10	0.98
18	1.01
Average	1.00

^∗^Molarity at each time point is based on the average of two sample preparations (*n* = 2).

This ^11^B qNMR method demonstrated excellent performance for the quantitation of boron trichloride in dichloromethane. It is anticipated that this method will be equally effective for boron trichloride in other organic solvents (e.g., heptane, toluene, etc.), although these were not evaluated in this study.

### 3.5. Applicability and Limitations of the Method

The qNMR method was developed to address a practical analytical need: the safe quantification and stability monitoring of bulk boron trichloride solutions in dichloromethane. The method has been successfully applied in routine stability studies and is particularly valuable in synthetic chemistry processes requiring accurate stoichiometric addition of boron trichloride.

The approach leverages the inherent selectivity of ^11^B NMR spectroscopy. Because ^11^B NMR detects only boron‐containing species, and because boron trichloride is converted quantitatively to a single and well‐resolved product (trimethyl borate), other organic components are effectively NMR‐silent and do not interfere with quantitation. On this basis, the method is expected to be applicable to more complex samples, provided that no additional boron‐containing species capable of producing overlapping ^11^B resonances are present. If other boron species were present, they would be expected to exhibit distinct chemical shifts and could be detected independently. This selectivity broadens the method’s utility in manufacturing environments where rapid and reliable assessment of boron trichloride concentration and stability is essential.

Although the method provides a safe, rapid, and reliable means of quantifying boron trichloride in dichloromethane, several limitations should be acknowledged. Validation demonstrated acceptable accuracy and precision at the concentrations evaluated (0.05, 0.1, and 0.2 M). Quantitation at substantially lower concentrations was not investigated and would require additional method optimization and validation. The method was specifically designed to support stability assessment of boron trichloride at a nominal concentration of 1.0 M, with a specification range of 0.9500–1.100, and performance at lower concentration levels was not considered critical for the intended application.

The method further assumes complete and quantitative conversion of boron trichloride to trimethyl borate, with no competing side reactions; this risk is considered minimal given the rapid and well‐established reaction of boron trichloride with methanol. Validation was performed exclusively in dichloromethane, and applicability to other organic solvents has not been evaluated. Because ^11^B relaxation times can vary with matrix composition and analyte concentration, the relaxation delay may require re‐optimization if these parameters change significantly. The use of an external standard also introduces potential variability associated with weighing, transfer, and handling. The external‐standard approach employed here is based on the PULCON method equation (1); if significant instrumental variability is observed, additional PULCON parameters (e.g., pulse length or temperature) may need to be incorporated. Finally, while the method minimizes direct handling risks, it does not eliminate all hazards associated with boron trichloride and requires access to a suitably equipped and qualified NMR facility capable of ^11^B analysis. In laboratories lacking such instrumentation, alternative approaches such as titration‐based methods may be required, subject to appropriate safety controls.

## 4. Conclusions

This study developed and validated a safe, accurate, precise, and robust ^11^B quantitative nuclear magnetic resonance (qNMR) method for quantifying boron trichloride in solution. By converting boron trichloride into trimethyl borate through a single‐step reaction and sample preparation within the same vial, the method ensures both efficiency and safety. The validation results confirmed the robustness and suitability of the analytical procedure for its intended purpose. This ^11^B qNMR technique effectively overcomes the challenges associated with the high reactivity and moisture sensitivity of boron trichloride, providing a dependable solution for its quantification in liquid samples.

## Author Contributions

All authors contributed equally to this work. All authors were involved in the conceptualization, methodology, investigation, data analysis, and writing and revision of the manuscript.

## Funding

No funding was received for this manuscript.

## Disclosure

All authors have read and approved the final manuscript.

## Conflicts of Interest

The authors declare no conflicts of interest.

## Data Availability

The data that support the findings of this study are available from the corresponding author upon reasonable request.

## References

[1] Gerrard W. and Lappert M. F. , Reactions of Boron Trichloride With Organic Compounds, Chemical Reviews. (1958) 58, no. 6, 1081–1111, 10.1021/cr50024a003.

[2] Kabalka G. W. , Wu Z. , and Ju Y. , The Use of Organoboron Chlorides and Bromides in Organic Synthesis, Journal of Organometallic Chemistry. (2003) 680, no. 1, 12–22.

[3] Soundararajan R. and Matteson D. S. , Hydroboration With Boron Halides and Trialkylsilanes, Journal of Organic Chemistry. (1990) 55, no. 8, 2274–2275, 10.1021/jo00295a004.

[4] Singh D. , Kumar G. , Dheer D. et al., BCl3-Mediated C–N, C–S, and C–O Bond Formation of Imidazo[1,2-a]pyridine Benzylic Ethers, ACS Omega. (2019) 4, no. 3, 4530–4539, 10.1021/acsomega.9b00035. PMC6648736 31459646

[5] Grosso A. D. , Helm M. D. , Solomon S. A. , Caras-Quintero D. , and Ingleson M. J. , Simple Inexpensive Boron Electrophiles for Direct Arene Borylation, Chemical Communications. (2011) 47, no. 46, 12459–12461, 10.1039/c1cc14226g. 22022702

[6] Kabalka G. W. and Wu Z. , Conversion of Aromatic Aldehydes to Gem-Dichlorides Using Boron Trichloride. A New Highly Efficient Method for Preparing Dichloroarylmethanes, Tetrahedron Letters. (2000) 41, no. 5, 579–581, 10.1016/s0040-4039(99)02065-1.

[7] Christie W. W. and Han X. , Christie W. W. and Han X. , Chapter 7-Preparation of Derivatives of Fatty Acids, Lipid Analysis, 2012, 4th edition, Woodhead Publishing, 145–158.

[8] Murphy G. M. , Separation of Boron isotope**s** , 1952, 1952 NNES III‐5, USAEC, Technical Information Service, Oak Ridge, Tennessee, US.

[9] Barbu B. N. , Kosak T. M. , Prins A. J. , Gillmore J. G. , and Korich A. L. , Fast Colorimetric Titration Protocol for Quantification of Boron Tribromide, Tetrahedron Letters. (2016) 57, no. 33, 3746–3748, 10.1016/j.tetlet.2016.07.017.

[10] Amaio A. M. , Siracusa R. A. , and Korich A. L. , Expanded Benchtop Protocol for the Colormetric Titration of Boron Trichloride and Bromide, Journal of Undergraduate Chemistry Research. (2023) 22, no. 3, 69–71.

[11] Schuele W. J. , Hazel J. F. , and McNabb W. M. , Analysis of Boron Tribromide and Its Addition Compounds, Analytical Chemistry. (1956) 28, no. 4, 505–507, 10.1021/ac50161a027.

[12] Hermanek S. , Boron-11 NMR Spectra of Boranes, Main-Group Heteroboranes, and Substituted Derivatives. Factors Influencing Chemical Shifts of Skeletal Atoms, Chemical Reviews. (1992) 92, no. 2, 325–362, 10.1021/cr00010a007.

[13] Aguilera-Sáez L. M. , Belmonte-Sánchez J. R. , Romero-González R. et al., Pushing the Frontiers: Boron-11 NMR as a Method for Quantitative Boron Analysis and Its Application to Determine Boric Acid in Commercial Biocides, Analyst. (2018) 143, no. 19, 4707–4714, 10.1039/c8an00505b. 30183032

[14] Pauli G. F. , Jaki B. U. , and Lankin D. C. , Quantitative 1H NMR: Development and Potential of a Method for Natural Products Analysis, Journal of Natural Products. (2005) 68, no. 1, 133–149, 10.1021/np0497301. 15679337

[15] Pauli G. F. , Chen S.-N. , Simmler C. et al., Importance of Purity Evaluation and the Potential of Quantitative 1H NMR as a Purity Assay, Journal of Medicinal Chemistry. (2014) 57, no. 22, 9220–9231, 10.1021/jm500734a. PMC4255677 25295852

[16] Salem Y. A. , El-Sayed S. M. , and El-Masry A. A. , Whiteness and Greenness Appraisal Approaches for Quantitative Determination of Recently Approved Combination of Indacaterol and Mometasone via Utilization of Nuclear Magnetic Resonance Technique, Sustainable Chemistry and Pharmacy. (2024) 39, 10.1016/j.scp.2024.101522.

[17] Bharti S. K. and Roy R. , Quantitative 1H NMR Spectroscopy, TrAC, Trends in Analytical Chemistry. (2012) 35, 5–26, 10.1016/j.trac.2012.02.007.

[18] Holzgrabe U. , Quantitative NMR Spectroscopy in Pharmaceutical Applications, Progress in Nuclear Magnetic Resonance Spectroscopy. (2010) 57, no. 2, 229–240, 10.1016/j.pnmrs.2010.05.001. 20633364

[19] Huang Y. , Su B.-N. , Ye Q. , Palaniswamy V. A. , Bolgar M. S. , and Raglione T. V. , Improving the Efficiency of Quantitative 1H NMR: An Innovative External Standard–Internal Reference Approach, Journal of Pharmaceutical and Biomedical Analysis. (2014) 88, 1–6, 10.1016/j.jpba.2013.07.043. 24013124

[20] Pinciroli V. , Biancardi R. , Visentin G. , and Rizzo V. , The Well-Characterized Synthetic Molecule: A Role for Quantitative 1H NMR, Organic Process Research & Development. (2004) 8, no. 3, 381–384, 10.1021/op0341925.

[21] Holzgrabe U. , Deubner R. , Schollmayer C. , and Waibel B. , Quantitative NMR spectroscopy—Applications in Drug Analysis, Journal of Pharmaceutical and Biomedical Analysis. (2005) 38, no. 5, 806–812, 10.1016/j.jpba.2005.01.050. 15893899

[22] Nishizaki Y. , Lankin D. C. , Chen S.-N. , and Pauli G. F. , Accurate and Precise External Calibration Enhances the Versatility of Quantitative NMR (qNMR), Analytical Chemistry. (2021) 93, no. 5, 2733–2741, 10.1021/acs.analchem.0c02967. PMC8046598 33481571

[23] Lee Y. , Matviychuk Y. , and Holland D. J. , Quantitative Analysis Using External Standards With a Benchtop NMR Spectrometer, Journal of Magnetic Resonance. (2020) 320, 10.1016/j.jmr.2020.106826. 33007501

[24] Coulibaly F. S. , Alnafisah A. S. , Oyler N. A. , and Youan B.-B. C. , Direct and Real-Time Quantification of Bortezomib Release From Alginate Microparticles Using Boron (11B) Nuclear Magnetic Resonance Spectroscopy, Molecular Pharmaceutics. (2019) 16, no. 3, 967–977, 10.1021/acs.molpharmaceut.8b00873. 30525665

[25] Watanabe R. , Sugai C. , Yamazaki T. et al., Quantitative Nuclear Magnetic Resonance Spectroscopy Based on PULCON Methodology: Application to Quantification of Invaluable Marine Toxin, Okadaic Acid, Toxins. (2016) 8, no. 10, 10.3390/toxins8100294. PMC5086654 27754382

[26] Ochoa J. L. , Germann S. , Conklin B. et al., The Effect of Tube Quality on Externally Calibrated Quantitative Nuclear Magnetic Resonance Analysis: How Bad can it be?, Magnetic Resonance in Chemistry. (2024) 62, no. 1, 4–10, 10.1002/mrc.5404. 37927097

[27] Wider G. and Dreier L. , Measuring Protein Concentrations by NMR Spectroscopy, Journal of the American Chemical Society. (2006) 128, no. 8, 2571–2576, 10.1021/ja055336t. 16492040

[28] Gerrard W. and Lappert M. F. , Interaction of Alcohols and Boron Trichloride, Journal of the Chemical Society, 1951, no. 0, 2545–2550.

[29] Kupai J. , Razali M. , Buyuktiryaki S. , Kecili R. , and Szekely G. , Long-Term Stability and Reusability of Molecularly Imprinted Polymers, Polymer Chemistry. (2017) 8, no. 4, 666–673, 10.1039/c6py01853j. PMC5361172 28496524

[30] Balz R. , Brändle U. , Kämmerer E. , Köhnlein D. , Lutz O. , and Nolle A. , 11B and 10B NMR Investigations in Aqueous Solutions, Zeitschrift für Naturforschung A. (1986) 41, no. 5, 737–742, 10.1515/zna-1986-0508.

[31] Momii R. K. and Nachtrieb N. H. , Nuclear Magnetic Resonance Study of Borate-Polyborate Equilibria in Aqueous Solution, Inorganic Chemistry. (1967) 6, no. 6, 1189–1192, 10.1021/ic50052a026.

[32] El-Masry A. A. , El-Wasseef D. R. , Eid M. , Shehata I. A. , and Zeid A. M. , Quantitative Proton Nuclear Magnetic Resonance Method for Simultaneous Analysis of Fluticasone Propionate and Azelastine Hydrochloride in Nasal Spray Formulation, Royal Society Open Science. (2021) 8, no. 7, 10.1098/rsos.210483. PMC8278066 34277026

[33] Malz F. and Jancke H. , Validation of Quantitative NMR, Journal of Pharmaceutical and Biomedical Analysis. (2005) 38, no. 5, 813–823, 10.1016/j.jpba.2005.01.043. 15893442

[34] Gödecke T. , Napolitano J. G. , Rodríguez-Brasco M. F. et al., Validation of a Generic Quantitative 1H NMR Method for Natural Products Analysis, Phytochemical Analysis. (2013) 24, no. 6, 581–597, 10.1002/pca.2436. PMC3990190 23740625

[35] (ICH), I.C.f.H. , ICH Harmonised Guideline: Validation of Analytical Procedures, 2023, no. R2.

